# Assessing the Psychosocial Impact of Acne Scars on Individuals With Pigmented Skin: A Multicenter Observational Study

**DOI:** 10.1155/drp/6657441

**Published:** 2025-09-27

**Authors:** Marwa Majzoub, Eleonore Goubeau, Vivien Moris

**Affiliations:** ^1^Department of Dermatology, Dijon University Hospital, Boulevard du Maréchal-de-Lattre-de-Tassigny, Dijon 21000, France; ^2^Department of Maxillofacial, Plastic, Reconstructive, Esthetic and Hand Surgery, Dijon University Hospital, Boulevard du Maréchal-de-Lattre-de-Tassigny, Dijon 21000, France

**Keywords:** acne scars, ECCA scale, Phototypes IV–VI, pigmented skin, psychosocial impact, quality of life (DLQI)

## Abstract

Acne scars, particularly in individuals with pigmented skin, can lead to significant psychosocial distress, yet the extent of this impact remains underexplored. This study aimed to assess the psychosocial effects of acne scars on patients with skin Phototypes IV–VI. This retrospective multicenter observational study involved 86 patients with acne scars who had previously consulted general practitioners. Scar severity was assessed using the Echelle d'évaluation Clinique des Cicatrices d'Acné (ECCA). Validated measures were used to evaluate psychological and quality-of-life impacts: the Patient Health Questionnaire-9 (PHQ-9) for depressive symptoms and the Dermatology Life Quality Index (DLQI) for quality of life. The study revealed that 62% of patients exhibited depressive symptoms according to the PHQ-9, and 84% reported a diminished quality of life according to the DLQI. A significant correlation was observed between acne scar severity and both psychosocial measures: ECCA and DLQI (*r* = 0.31, *p*=0.003), and ECCA and PHQ-9 (*r* = 0.27, *p*=0.010). Many participants had modified their clothing and daily activities due to their scars. The findings illustrate the profound psychosocial burden of acne scars, with a notable percentage of individuals experiencing depressive symptoms and reduced quality of life. The strong correlation between scar severity and psychosocial outcomes emphasizes the need for early, comprehensive care that addresses both dermatological and psychological aspects.

## 1. Introduction

Acne is a chronic inflammatory disease of the pilosebaceous unit that predominantly occurs during adolescence. It is one of the most widespread dermatological conditions, affecting a significant portion of adolescents across the globe. Acne vulgaris is recognized as a multifactorial chronic inflammatory disorder of the pilosebaceous unit, representing the most common skin disease that affects approximately 85% of adolescents in Western populations [1], with moderate to severe forms representing 20% of these individuals [2]. Acne scars are very prevalent and are found in 95% of men and women with acne, particularly among those who have experienced moderate to severe acne lesions. The incidence of acne scars tends to be greater in patients with a history of more severe acne conditions [3].

Acne can lead to significant psychological distress during both adolescence and adulthood. The psychological and social effects of acne scars are insufficiently studied in the scientific literature. Acne significantly impacts social and psychological well-being, particularly when it affects visible areas such as the face and body, leading to decreased self-confidence, fear of judgment, and reluctance in social and friendly relationships [4]. Diminished self-esteem can negatively affect intimate, romantic, and sexual relationships while also leading to poor academic performance and an increased risk of unemployment later in life. [5]. Studies have shown that acne can lead to depressive symptoms and an increased risk of suicidal thoughts, with suicidal ideation found in 6%-7% of acne patients [6]. The severity of acne and the extent of scarring can exacerbate these psychological effects, underscoring the importance of effective acne management and support for individuals with acne scars [7, 8].

**Figure 1:**
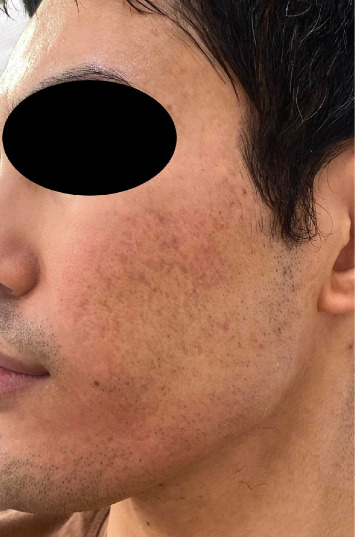
35-year-old patient Phototype 4 with hyperpigmented acne scars.

For patients with dark skin, acne scars frequently lead to postinflammatory hyperpigmentation (PIH) (Figure 1), hypertrophy, or keloid scarring. These scars are more visible and exacerbate the psychosocial impact. These individuals are more prone to PIH due to their higher melanin content, which intensifies the skin's response to the inflammatory processes of acne [9].

The aim of this study was to assess the psychosocial impact of acne on patients with pigmented skin (phototype ≥ 4) who have acne scars.

## 2. Materials and Methods

This retrospective multicenter observational study included adult patients from Seine-Saint-Denis and Quetigny with Phototypes 4, 5, or 6 who presented with acne scars and had previously consulted general practitioners (GPs) who used the same medical approach for acne patients using questionnaires.

Seine-Saint-Denis is a highly diverse and multicultural department in the Île-de-France region. A significant portion of its population is of African descent, contributing to a higher prevalence of darker skin phototypes in the study population [10]. This demographic context supports the relevance of focusing on patients with skin Phototypes IV–VI. Patients with chronic dermatological conditions such as vitiligo, eczema, atopic dermatitis, and psoriasis, as well as those with psychiatric histories or who refused to consent, were excluded.

Medical files were collected in three locations: first, at the Salvador Allende Municipal Health Center in La Courneuve, and then in GPs' offices in Noisy-le-Grand and Quetigny.

The primary endpoint of the study was the psychosocial impact of acne scars, which was assessed through two self-report questionnaires: the Patient Health Questionnaire (PHQ-9) for measuring depression diagnosis and severity [11] and the Dermatology Life Quality Index (DLQI) for evaluating the impact of skin diseases on patients' quality of life [12].

The secondary endpoint focused on analyzing the correlation between the severity of acne scars, as evaluated by the Echelle d'évaluation Clinique des Cicatrices d'Acné (ECCA) score, and the psychosocial impact, as measured by the same three questionnaires.

### 2.1. Score

The ECCA score, assessed by the GP during consultation, consists of six items, each representing a specific type of acne scar [13]. Each item is assigned a quantitative score ranging from 0 to 4, reflecting the number of scars, and a weighting factor between 15 and 50.

The PHQ-9 is a tool that assesses the presence and severity of depressive symptoms. It quantifies depression severity with total scores ranging from 0 to 27.

The DLQI is a generic quality-of-life measure for patients with dermatological conditions. It consists of questions that are scored to produce a total ranging from 0 to 30. A higher score indicates a greater impact on quality of life.

GPs routinely use these different questionnaires in their daily practice. The results of the ECCA score and the two psychosocial impact questionnaires (PHQ-9 and DLQI) were documented in the acne patients' files. In this study, although data collection was retrospective, the PHQ-9 and DLQI questionnaires were administered prospectively as part of routine clinical practice by GPs, who systematically informed patients that their responses might be used for future research in an anonymized fashion. Written informed consent was thus obtained at the time of questionnaire administration, prior to the data being compiled for this retrospective analysis.

This study was reviewed and deemed exempt by the Direction de la Recherche Clinique et de l'Innovation (DRCI) at the University Hospital of Dijon Institutional Review Board (IRB) as it involved retrospective data analysis and did not include identifiable patient information. All procedures were conducted in accordance with the ethical standards of the DRCI Ethics Committee and the Declaration of Helsinki (2013 revision).

### 2.2. Statistics

Statistical analyses were performed using GraphPad Prism 7 software. Normal distribution was evaluated by the Kolmogorov–Smirnov test and graphic QQ plots. The correlation calculation was performed using Spearman's method, with a *p* value less than 0.05 considered significant.

## 3. Results

Eighty-six patients were included between January and June 2022. Women accounted for 77% of the participants. The average age was 31 years, ranging from 17 to 64 years. The age group of 20–40 years was the most represented, with 56 patients (65%). The characteristics of the patients and their scars are presented in Table 1.

Phototype 4 was the most common, accounting for 51% of the population, while Phototype 6 comprised 12% of the population (Table 1). The face was the area most affected by acne scars, with 61 occurrences, followed by the neck and back, the arms and shoulders, and finally, the chest. Notably, 36% of the patients had multiple affected areas.

The majority of the scars were atrophic, accounting for 87%, with an even distribution among the scar types: 37% were U-shaped (rolling scars), 32% were V-shaped (ice pick scars), and 32% were M-shaped (boxcar scars). There were 7 patients with keloid scars and another 7 with hypertrophic scars identified during the clinical examination, accounting for 13% of the total scars (Table 2). Of all the lesions observed, 88% exhibited hyperpigmented acne scars.

The ECCA score was assessed for each patient included in the study, with a median of 30 and a range of 195 [15–210].

### 3.1. Primary Outcome

The primary outcome measure was the psychosocial impact of acne scars, evaluated using the PHQ-9 and DLQI questionnaires (Table 3). Among the 86 patients included in the study, only 16% reported no psychosocial impact. Specifically, 62% of the patients showed depressive symptoms as assessed by the PHQ-9, and 84% experienced an impact on their quality of life according to the DLQI.

The patients' depressive states were evaluated using the PHQ-9, yielding a median score of 5.5 (range: 0–25). Nearly half of the patients (48%) experienced mild to moderate depression, while 5 patients (6%) experienced moderately severe to severe depression, as indicated by a PHQ-9 score above 15 (Table 3).

Quality of life was assessed using the DLQI. The median DLQI score was 4 (range: 0–23), with a majority of patients (84%) experiencing an impact on their quality of life due to acne scars (Table 3). For 16% of the patients, acne scars had no impact on their quality of life.

According to the DLQI analysis, 59% of patients felt the need to alter their clothing to conceal their scars, while 51% reported that their leisure activities were affected by acne scars. In addition, 66% of patients noted that the skin treatments and techniques used to conceal acne scars resulted in a daily loss of time. On a positive note, the majority of patients (61%) reported no inconvenience in performing activities such as shopping or household chores. Similarly, approximately 60% of the participants experienced no difficulty engaging in sports, working, or maintaining their personal or sexual relationships.

A subgroup analysis examined gender differences in the correlation between acne scar severity and quality of life. Among women, the correlation between ECCA and DLQI was moderate (*r* = 0.38), whereas it was minimal in men (*r* = 0.007). A Fisher's r-to-z transformation comparing these correlations yielded a *z* value of 1.42 (*p*=0.157), indicating that the difference was not statistically significant.

### 3.2. Secondary Outcome

The secondary outcome measure involved analyzing the correlation between the severity of acne scars (ECCA score) and psychosocial impact, as assessed by two questionnaires: the PHQ-9 and DLQI.

A statistically significant correlation was found between the ECCA score and quality of life. The strongest correlation was between the ECCA score and the DLQI, with *r* = 0.31 and a confidence interval (CI) of 0.10–0.49, *p*=0.003; this was followed by the ECCA/PHQ-9 correlation, with *r* = 0.27, CI = 0.063–0.46, and *p*=0.010.

## 4. Discussion

Acne is an inflammatory dermatological disease that disappears over time in some patients. However, the psychological impact of acne scars persists because they tend to worsen due to the natural aging process of the skin [14]. The psychological effect of acne and its scars has been well demonstrated to have an emotional impact comparable to that of some chronic diseases, such as diabetes, asthma, epilepsy, or arthritis [15].

This study specifically focused on acne scars in patients with pigmented skin: Phototypes IV, V, and VI. It was a retrospective multicenter study conducted in various regions (Ile-de-France and Burgundy). Eighty-six patients were included. The results of the questionnaires were found in the medical files of the patients. These doctors used the same medical approach for acne patients after attending a medical conference on dermatology in general practice.

The primary aim of this study was to assess the psychosocial burden of acne scars using validated self-report measures. Findings indicate that a majority of patients experienced emotional or quality-of-life impacts related to their scarring, with depressive symptoms commonly reported. Quality of life was commonly affected by acne scars, with many patients reporting changes in daily behavior, such as adjusting clothing or avoiding certain activities. Despite these impacts, most were still able to maintain social and professional engagement without major functional limitations.

Indeed, Neirita Hazarika and Archana reported that 66% of patients with acne scars experience psychosocial disorders ranging from moderate to severe, as assessed by the DLQI [16]. Behnam et al. demonstrated the negative influence of acne and its scars on the psychological state of patients, with the presence of the following symptoms: obsession, depression, anxiety, hostility, phobia, and paranoia [17]. Fried et al. studied 97 subjects suffering from acne scars; 85.4% of the study subjects revealed that they were dissatisfied with looking at themselves in the mirror, and 84.4% felt less attractive due to their skin problems [18].

Our study identified a significant link between the severity of acne scars, as measured by the ECCA score, and their impact on quality of life, highlighted by correlations with the DLQI and PHQ-9. The strongest correlation was observed between the ECCA score and the DLQI (*r* = 0.31, *p*=0.003), indicating that more severe acne scars tend to worsen patients' quality of life. Concerning the gender subgroup analysis, the stronger ECCA–DLQI correlation in women suggests a greater psychosocial burden, potentially reflecting gender-based differences in the perceived impact of visible skin conditions. This pattern aligns with the evidence that women are more affected by acne-related distress [19], though the difference was not statistically significant, possibly due to the limited number of male participants.

A slightly less pronounced but still significant correlation was found with the PHQ-9 (*r* = 0.27, *p*=0.010), indicating a notable psychological impact associated with scar severity.

These correlations are also described in the literature; Hazarika found a significant correlation between the presence of acne scars and the impairment of quality of life assessed by the DLQI, with *p*=0.05 for Question Q3 of daily activities and Question Q8 of interpersonal problems (*p*=0.028) [16]. Vilar et al. reported a correlation between the severity of acne and deterioration of quality of life [20]. Similarly, Tasoula et al. noted that patients with severe acne had weakened self-esteem with a deterioration of their body image perception and relational difficulties [21].

Families and friends of patients frequently view acne and its scars as minor cosmetic issues, overlooking their significant psychosocial implications. GPs too often underestimate these concerns, not fully recognizing the lasting emotional and social challenges they pose. Our study revealed that acne scars have the potential to cause the same psychosocial difficulties as acne itself, yet the scars remain with the individual for life. The most alarming aspect is the psychological impact, notably depression, which poses serious risks, including social isolation and the possibility of suicide.

Preventing acne scars is possible with timely and effective treatment in adolescence. It is recommended that a dermatologist start appropriate treatments, including topical or systemic options such as isotretinoin, as needed [22, 23]. For existing acne scars, treatments such as PRP therapy, microneedling, fillers, laser therapy, or chemical peels can help diminish scar visibility [14]. Avoiding the onset of inflammatory acne through proactive prevention and early intervention is key to preventing scarring. In this effort, the role of a GP in early detection and management is vital. In France, the delay for consulting a dermatologist is between 61 and 126 days, making it the second most difficult specialist for patients to access [24]. That is why GPs are increasingly coming into contact with acne patients. They need to be aware of this condition and the psychological risks associated with it and its scars.

### 4.1. Limitations

A key limitation of our study is the absence of a nonacne control group, which limits our ability to determine whether the psychological distress observed is specifically attributable to acne scarring, as participants may have had other unrelated factors contributing to elevated DLQI and PHQ-9 scores. In a hospital-based case-control study, Nadim et al. found that individuals without acne had significantly better quality of life [25], with a mean DLQI score of 1.02 ± 1.4 compared to 8.2 ± 5.0 in acne patients, reinforcing the substantial psychosocial impact of acne-related skin conditions.

The PHQ-9 assesses depressive symptoms over the past 15 days, which may introduce temporal bias and capture distress unrelated to acne scars, potentially confounding the results.

In conclusion, this study demonstrates that the impact of acne scars extends beyond dermatological concerns, significantly affecting psychological well-being and quality of life. Early and comprehensive management is essential, given the emotional burden associated with scarring. A multidisciplinary approach involving dermatologists and mental health professionals is crucial not only to initiate early acne treatment and scar management but also for identifying psychosocial distress and facilitating appropriate psychological support with referral if needed.

## Figures and Tables

**Table 1:** Patient's characteristics.

Patient characteristics	Number (%)
Sex	
Woman	67 (77%)
Man	19 (23%)
Age	
17–20 years old	14 (16%)
20–40 years old	56 (65%)
> 40 years old	16 (19%)
Tobacco consumption	
Nonsmoking	59 (68%)
5–10 cigarettes/day	17 (20%)
10–20 cigarettes/day	10 (12%)
Skin type	
Phototype 4	44 (51%)
Phototype 5	32 (37%)
Phototype 6	10 (12%)

**Table 2:** Distribution and types of scars.

Scars description	Number (%)
Scar distribution	
Face	61 (54%)
Neck/back	28 (25%)
Arm/shoulder	12 (11%)
Cleavage	11 (10%)
Scar type	
Atrophic	92 (87%)
U shape	34
V shape	29
M shape	29
Hypertrophic	14 (13%)
Keloid	7
Hypotrophic	7

**Table 3:** Results of the PHQ-9 and DLQI questionnaires.

PHQ-9	No depression (0–4)	Mild depression (5–9)	Moderate depression (10–14)	Moderately severe depression (15–19)	Severe depression (20–27)
	33 (38%)	27 (31%)	21 (25%)	3 (4%)	2 (2%)

**DLQI**	**No effect (0-1)**	**Small effect (2–5)**	**Moderate effect (6–10)**	**Very large effect (11–20)**	**Extremely large effect (21–30)**

	14 (16%)	34 (40%)	13 (15%)	23 (27%)	2 (2%)

## Data Availability

All data are available upon request from the first author, Dr. Marwa Majzoub.
